# *Magnolia kobus* DC. suppresses neointimal hyperplasia by regulating ferroptosis and VSMC phenotypic switching in a carotid artery ligation mouse model

**DOI:** 10.1186/s13020-024-01051-4

**Published:** 2025-01-03

**Authors:** Jong Min Kim, Yiseul Kim, Hyun-Jin Na, Haeng Jeon Hur, Sang Hee Lee, Mi Jeong Sung

**Affiliations:** Aging and Metabolism Research Group, Korea Food Research Institute, Wanju‑gun, 55365 Republic of Korea

**Keywords:** *Magnoliakobus* DC., Ferroptosis, Neointimal hyperplasia, VSMC, Vascular disease

## Abstract

**Background:**

*Magnolia kobus* DC (MO), as a plant medicine, has been reported to have various physiological activities, including neuroprotective, anti-inflammatory, and anti-diabetic effects. However, vascular protective effects of MO remain incompletely understood. In this study, we evaluated the vascular protective effect of MO against ferroptosis in a carotid artery ligation (CAL)-induced neointimal hyperplasia mouse model and in aortic thoracic smooth muscle A7r5 cells.

**Methods:**

This study was conducted to estimate the vascular protective effects of MO by systematically measuring histopathological analysis and western blot analysis in CAL animal model. In vitro protective effects of MO were evaluated by estimating cell viability, reactive oxygen species (ROS) content, glutathione (GSH) levels, lipid peroxidation, mitochondrial morphological change, cell proliferation, migration, western blot analysis, and qRT-PCR against erastin (Era)-induced A7r5 cells.

**Results:**

MO intake significantly improved neointimal formation, inhibited ferroptosis and vascular smooth muscle cell (VSMC) phenotypes, and ameliorated the antioxidant system of carotid artery tissues. In addition, MO treatment effectively ameliorated Era-induced ferroptotic cytotoxicity, including cellular death, ROS production, and cell migration status. MO treatment also suppressed proliferation and migration in Era-induced A7r5 cells. MO considerably regulated Era-induced abnormal mechanisms related to ferroptotic changes, VSMC phenotype switching, and the ROS scavenging system in A7r5 cells.

**Conclusion:**

MO has the potential for use as a functional food supplement, nutraceutical, or medicinal food, with protective effects on vascular health by regulating ferroptosis and VSMC phenotypic switching.

## Introduction

Ferroptosis is a form of cell death associated with the accumulation of iron ions and lipid peroxidation [1]. This pathway is distinct from other forms of cell death and is primarily associated with cell damage and diseases, particularly those affecting the vascular system [2]. The role of ferroptosis, in vascular smooth muscle cells (VSMCs) and endothelial cells, has potential implications for vascular health and disease progression [3]. Ferroptosis involves excessive accumulation of iron, inflammatory cytokines, and oxidative stress, which promote lipid peroxidation, particularly of phospholipids, which are essential components of cell membranes [4]. This process increases cell membrane instability, leading to cell damage and death [5]. Especially, the xCT protein plays an important role in transporting cysteine into cells, contributing to the synthesis of glutathione (GSH), which is a key antioxidant involved in protecting cells from oxidative stress [6]. However, during ferroptosis, the reduced activity of the xCT protein leads to a shortage of cysteine supply in the VSMCs, resulting in decreased GSH levels, which maintains the balance between pro-oxidant and antioxidant systems in VSMCs [7]. This makes the cells more vulnerable to oxidative stress, further promoting lipid peroxidation and iron accumulation [8]. In addition, the accumulation of iron and lipid peroxidation can damage endothelial cells and VSMC within blood vessels and increase the instability of the vascular wall [9]. This can increase the risk of atherosclerosis, blood clot formation, and vascular diseases [10]. Furthermore, ferroptosis is related to the development and progression of cardiovascular diseases, such as myocardial infarction and stroke [11]. While there has been significant research focusing on the impact of ferroptosis on endothelial function and neointimal formation, there is a lack of comprehensive studies investigating the specific effects on VSMCs and how these effects contribute to vascular remodeling and disease. Understanding the mechanisms by which ferroptosis influences VSMC phenotype and function is crucial for developing therapeutic strategies aimed at mitigating vascular damage and preventing disease progression.

*Magnolia kobus* DC*.* (MO), a plant medicine officially registered in the Asian National Pharmacopoeia and known as Xhin-Yi, is made from the dried flower buds of the magnolia tree, which is mainly found in East Asia [12]. MO has been reported to possess various physiological activities, including neuroprotective, anti-inflammatory, and anti-diabetic effects [13, 14]. MO contains various phenylpropanoid, such as *p*-hydroxybenzoic acid, chlorogenic acid, *p*-coumaric acid, and kaempferol, essential oils, containing citral, trans-anethole, and methyl chavicol, and alkaloids, such as salicifoline, sinapyl alcohol, syringing, and phosphatidylcholine [15, 16]. Various studies have reported a correlation between neointimal hyperplasia and ferroptosis, however, there are few studies on the ameliorating effects of MO. Therefore, in this study, the ameliorating effect of the MO extract was evaluated to identify the composition of bioactive compounds and to determine whether it regulates vascular ferroptosis via VSMC phenotype switching in a carotid artery ligation (CAL) mouse model and A7r5 cells.

## Materials and methods

### Sample preparation

MO cultivated in Jeollabuk-do (Republic of Korea) was purchased from a local market (Jeonju, Republic of Korea). The MO was ground to powder form and stored at −  20 °C. The ground samples were extracted with 50-fold volumes of 50% ethanol at 40 °C for 24 h. The extracted samples were filtered through Whatman No. 2 filter paper (Whatman International Limited, Kent, UK) and concentrated using a vacuum rotary evaporator.

### Ultra performance liquid chromatography quadrupole-time-of-flight mass spectrometry (UPLC-QTOF/MS^E^) with electrospray ionization (ESI)

To identify the physiologically bioactive compounds, the MO dissolved in 50% methanol was analyzed using UPLC-Q-TOF/MS^E^ (Xevo G2-S, Waters Corp., Milford, MA, USA) with a BEH C_18_ column (100 × 2.1 mm, 1.7 μm; Waters Corp.). The mobile phases were solvent A (0.1% formic acid in distilled water) and solvent B (0.1% formic acid in acetonitrile). The conditions used for the ESI source were as follows: ramp collision energy, 20–45 V; desolvation temperature, 400 °C; source temperature, 40 °C; capillary voltage, 2.5 kV; cone voltage, 40 V; mass range, 50–1500 m/z. The UPLC-Q-TOF/MS^E^ results were analyzed using data analysis software (Waters MassLynx™, Waters Corp.).

### Animal experimental and surgical procedures

C57BL/6 (6-weeks-old, male) mice were purchased from Oriental Biotechnology (Daejeon, South Korea). The experimental mice were maintained under standard housing conditions (temperature of 22 ± 2 °C; humidity of 50 ± 60%; 12/12 h light/dark cycle) and allowed free access to food and water. The animal experimental procedures were approved by the Institutional Animal Care and Use Committee (IACUC) of the Korea Food Research Institute (certificate number: KFRI-M-23001) and performed in accordance with the institutional guidelines established by the Ethical Committee. During the experimental surgery, animal suffering was minimized, and the mice were anesthetized with 2.5% isoflurane. To induce neointimal hyperplasia, the common right carotid artery was ligated, as previously described [17], Briefly, the right common carotid artery proximal to the distal bifurcation was ligated using a 6.0 silk suture. The mice were randomly assigned to four groups: non-CAL with vehicle (SHAM group, n = 10), CAL with vehicle (CAL group, n = 10), CAL with MO administration (MO group, n = 10, 100 mg/kg/day), and CAL with ferrostatin-1 (Fer-1) administration as an inhibitor of ferroptosis (Fer-1 group, n = 10, 1 mg/kg/day). The mice were allowed to adapt for 3 days and were provided pure drinking water. MO or Fer-1 was administered via oral gavage for 4 weeks. The carotid artery proximal to the ligation region was fixed with 4% formaldehyde for histopathological analysis.

### Histopathological analysis

Fixed carotid arteries were dehydrated and embedded in paraffin. The organs were sectioned at 4 μm thickness, dewaxed, and rehydrated. The sliced tissues were stained with hematoxylin and eosin (H&E) and subjected to immunohistochemistry for proliferating cell nuclear antigen (PCNA). To estimate neointimal formation, stained slides were analyzed under a panoramic microscope (Olympus). The sectioned tissues were stained with H&E, and the intimal and medial areas of the vessels were defined as the area between the luminal circumference and the internal elastic lamina and the area between the internal and external elastic lamina, respectively. To conduct PCNA staining, the sliced tissues were incubated with the anti-PCNA antibody (13110S, Cell Signaling Technology, Danvers, MA, USA, 1:100) at 4 °C overnight. After incubation with secondary antibodies (Cell Signaling Technology), the PCNA scores were calculated in randomly selected 3 fields and visualized with 3,3′-diaminobenzidine (Dako, Glostrup, Denmark) using ImageJ software (NIH, Bethesda, ME, USA).

### Cell culture

A7r5 cells derived from the aortic thoracic smooth muscle of rats were purchased from the American Type Culture Collection (ATCC, Manassas, VA, USA). The A7r5 cells were cultured in Dulbecco’s modified Eagle’s medium (ATCC) containing 10% fetal bovine serum and penicillin (100 U/mL)/streptomycin (100 μg/mL) (ATCC) at 37 °C in a humidified 5% CO_2_ incubator (Forma steri-cycle i106, Thermo Fisher Scientific, Waltham, MA, USA).

### Western blot analysis

Carotid artery tissue and A7r5 cells were lysed in lysis buffer (9803S; Cell Signaling Technology). Protein lysates were separated using sodium dodecyl sulfate–polyacrylamide gel electrophoresis and transferred to polyvinylidene fluoride membranes. The membranes were then incubated with 3% bovine serum albumin in Tris-buffered saline and 0.1% Tween-20 at 25 °C room temperature. The blocked membranes were reacted with primary antibodies overnight at 4 °C. The primary antibody information is as follows: xCT (ab175186, Abcam Cambridge, UK), glutathione peroxidase 4 (GPX4; ab125066, Abcam), prostaglandin-endoperoxide synthase 2 (Ptgs2; ab15191, Abcam), smooth muscle 22α (SM22α; ab14106, Abcam), alpha smooth muscle actin (αSMA; ab14106, Abcam), osteopontin (OPN; ab166709, Abcam), nuclear factor erythroid-2-related factor 2 (Nrf2; sc-365949, Santa Cruz Biotechnology, Dallas, TX, USA), NADPH oxidase (NOX) 4 (ab109225, Abcam), superoxide dismutase 1 (SOD1; sc-17767, Santa Cruz Biotechnology), calponin-1 (17819S, Cell Signaling Technology, Danvers, MA, USA), soluble epoxide hydrolase (sEH; sc-166961, Santa Cruz Biotechnology), and β-actin as an internal control (sc-47778, Santa Cruz Biotechnology). Membranes were then incubated with horseradish peroxidase-conjugated goat anti-rabbit and anti-mouse secondary antibodies (Cell Signaling Technology). The luminescence of the bands was detected using enhanced chemiluminescence detection reagents (Cytiva, Marlborough, MA, USA), and the protein densities were calculated using ImageJ software (NIH).

### Quantitative real-time PCR (qRT-PCR)

RNA was isolated from A7r5 cells using an RNeasy RNA isolation kit (Qiagen, Valencia, CA, USA). The concentration of RNA extracted from the carotid artery was measured using a NanoDrop Spectrophotometer (Thermo Fisher Scientific). The extracted RNA was reverse-transcribed into cDNA using an iScript cDNA synthesis kit with random primers. The synthesized cDNA was used for qRT-PCR using a SYBR Green PCR Master Mix Kit (Bio-Rad, Hercules, CA, USA). The following primer sequences (5′−3′) were used in this study: *Acta2* (αSMA) forward (F), CAT CCG ACC TTG CTA ACG GA; *Acta2* (αSMA) reverse (R), AGA GTC CAG CAC AAT ACC AGT; *Cnn1* (calponin-1) F, GCC CAG AAA TAC GAC CAC CA; *Cnn1* (calponin-1) R, TGG AGC TTG TG ATA AAT TCG CA; *Myh11* (SM-MHC) F, ATC ACG GGG GAG CTG GAA AA; *Myh11* (SM-MHC) R, AAT GAA CTT GCC AAA GCG GG; *Sod* (SOD) F, GCG TCA TTC ACT TCG AGC AG; *Sod* (SOD) R, GGT CTC CAA CAT GCC TCT CT; *Cat* (CAT) F, ACA CTT TGA CAG AGA GCG GA; *Cat* (CAT) R, TTT CAC TGC AAA CCC ACG AG; *Nox1* (NOX1) F, CAC TCC CTT TGC TTC CTT CT; *Nox1* (NOX1) R, GCA CCC GTC TCT CTA CAA ATC; *Nox2* (NOX2) F, CTT TAG CAT CCA TAT CCG CAT T; *Nox2* (NOX2) R, GAC TGG TGG CAT TGT CAC AAT A; *Gapdh* (glyceraldehyde 3-phosphate dehydrogenase, GAPDH) F, ATT GTC AGC AAT GCA TCC TG; *Gapdh* (GAPDH) R, ATG, GAC TGT GGT CAT GAG CC. GAPDH was used as an internal control.

### Cell viability

A7r5 cells (1 × 10^4^ cells/well) were seeded into a 96-well plate and incubated for 24 h. Seeded cells were treated with erastin (Era) and MO/Fer-1 for 24 h. Cell viability was measured using a CCK-8 assay kit (DONGIN LS CO, Hwaseong, Republic of Korea), according to the manufacturer’s protocol, at a wavelength of 450 nm using a microplate reader (Spectra MAX 190; Molecular Devices, San Jose, CA, USA).

### Reactive oxygen species (ROS) content

A7r5 cells (1 × 10^4^ cells/well) were seeded in black 96-well plates and incubated for 24 h. The seeded cells were treated with Era and MO/Fer-1 for 24 h. ROS content in A7r5 cells was detected using dichlorodihydrofluorescein diacetate (10 μM) for 30 min at 37 °C in the dark. Fluorescent signals were detected at 450 nm using a fluorescence microplate reader (Molecular Devices).

### GSH levels

A7r5 cells (1 × 10^4^ cells/well) were seeded into a 96-well plate and incubated for 24 h. The seeded cells were treated with Era and MO/Fer-1 for 24 h. GSH levels in A7r5 cells were measured using a GSH detection kit (DoGenBio, Seoul, Republic of Korea), according to the manufacturer’s protocol, at a wavelength of 412 nm using a microplate reader (Molecular Devices).

### Lipid peroxidation

A7r5 cells were seeded in 6-well plates (1 × 10^5^ cells/well). The seeded cells were treated with Era and MO/Fer-1 for 16 h. Intracellular lipid peroxidation was evaluated with C11-BODIPY581/591 (5 μM, Thermo Fisher Scientific) under dark incubation for 30 min at 37 °C. For fluorescence measurements, cells were washed twice with phosphate-buffered saline and analyzed at excitation/emission wavelength of 581/591 nm using a microplate reader (Molecular Devices).

### Transmission electron microscopy (TEM) analysis

A7r5 cells were plated in 60 mm culture dishes. Seeded cells were treated with Era and MO/Fer-1 for 24 h. Cells were digested and fixed with 2% glutaraldehyde and 1% osmium tetroxide. Cell samples were then cut into ultrathin sections, stained with 2% uranyl acetate, dehydrated, embedded, and stained with lead citrate. Images were acquired with a transmission electron microscope (HT7700, Hitachi, Ibaraki, Japan).

### Cell proliferation

A7r5 cells (3 × 10^3^ cells/well) were incubated in 96-well plates for 24 h and treated with Era and MO/Fer-1. After 24 h, cell proliferation was measured using a BrdU assay kit (Merck Millipore, Burlington, MA, USA), according to the manufacturer’s protocol, at a wavelength of 450 nm using a microplate reader (Molecular Devices).

### Scratch wound-healing assay

A7r5 cells were seeded in 6-well plates (5 × 10^5^/well). The seeded cells were scraped using a sterile pipette tip at the center and treated with Era and MO/Fer-1. Each well was observed 0 and 24 h after scratching. Wound-healing areas were calculated using ImageJ image analysis software (NIH).

### Transwell migration

The migration assay was evaluated using a Transwell chamber plate with 8 μm pore size (Costar; Corning Incorporated, Corning, NY, USA). A7r5 cells seeded into the upper chambers (2 × 10^4^/well) were treated with Era and MO/Fer-1 for 24 h. Unmigrated cells were eliminated from the upper side using a clean cotton swab. Cells that migrated to the bottom were fixed using 4% paraformaldehyde and stained with 0.1% crystal violet for 30 min. Migrating cells were counted in randomly selected 3 fields.

### Statistical analysis

The experimental data were presented as the mean ± standard error of the mean (SEM). Comparisons between groups were performed using one-way analysis of variance, followed by Tukey’s post-hoc analysis using GraphPad Prism software (version 9.0; Inc., La Jolla, CA, USA). Statistical significance was set at *P* < 0.05, 0.01, and 0.001.

## Results

### MO contains various bioactive compounds

**Fig. 1:**
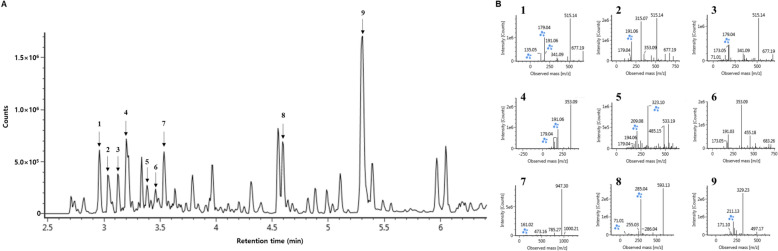
Identification of physiological compounds in MO. **A** UPLC-Q-TOF/MS^E^ in negative ion mode. **B** Fragments of identified compounds in MO. **1**, **2**, and **3**, 4-O-(3'-O-D-glucopyranosyl)-caffeoyl quinic acid isomers; **4**, Chlorogenic acid; **5**, syringin 4-O-β-glucoside; **6**, patuletin-3-O-(4''-O-acetyl-α-L-rhamnopyranosyl)−7-O-(2'''-O-acetyl-α-L-rhamnopyranoside); **7**, Magnoloside C; **8**, Kaempferol 3-(p-coumaroyl-glucoside); **9**, pinellic acid

The UPLC-QTOF-MS^E^ analysis was conducted to qualitatively identify the physiological compounds in MO (Fig. 1). The obtained MS spectra in negative ion mode [M − H]^−^ were analyzed using the online database UNIFI 1.8.2 (Waters Corp.) and other previous studies, and the separated peaks were tentatively identified as 4-O-(3'-O-D-glucopyranosyl)-caffeoyl quinic acid isomers (Fig. 1B; 1–3), chorogenic acid (Fig. 1B; 4), syringin 4-O-β-glucoside (Fig. 1B; 5), patuletin-3-O-(4''-O-acetyl-α-L-rhamnopyranosyl)−7-O-(2'''-O-acetyl-α-L-rhamnopyranoside) (Fig. 1B; 6), magnoloside C (Fig. 1B; 7), kaempferol 3-(p-coumaroyl-glucoside) (Fig. 1B; 8), and pinellic acid (Fig. 1B; 9).

### MO suppresses neointimal hyperplasia against CAL model

**Fig. 2:**
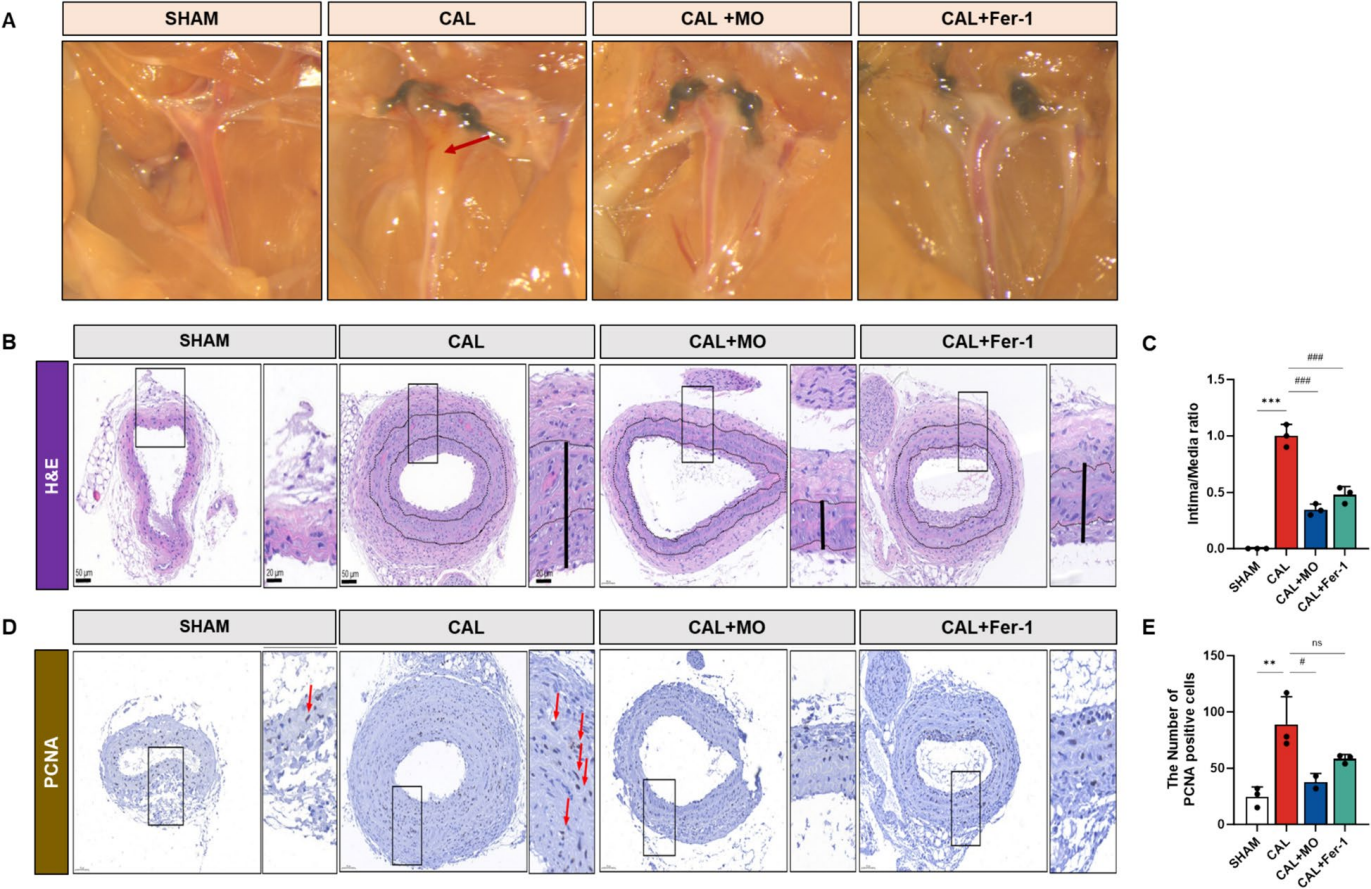
MO suppresses ligation-induced neointima formation in mouse carotid arteries. **A** Mouse carotid artery arch; arrow indicates plaque. **B** Carotid artery stained with H&E. **C** Intima/media ratio in carotid artery. **D** Carotid artery stained with proliferating cell nuclear antigen (PCNA). **E** The number of PCNA-positive cells in the carotid artery. Results are represented as mean ± SEM. ** *P* < 0.01, *** *P* < 0.001 compared to the SHAM group. ^#^ *P* < 0.05, ^##^ *P* < 0.01, ^###^ *P* < 0.001 compared to the CAL group

To investigate whether ferroptosis mediates neointimal formation, a well-known vascular remodeling model was established by ligating the carotid arteries in mice. After ligation treatment with the sample intake, the neointimal status of the carotid artery was observed by microscopy and histological analysis using H&E staining (Fig. 2A–C). This result confirmed the production of neointima and an increase in the intima/media ratio by ligation. However, the intake of MO and Fer-1, an inhibitor of ferroptosis, ameliorated the neointimal status. Histological analysis of PCNA staining showed that CAL stimulated the proliferation of carotid artery tissues. However, the consumption of MO and Fer-1 inhibited vascular proliferation by reducing the number of PCNA-positive cells (Fig. 2D and E).

### MO inhibits ferroptosis and phenotypes change of VSMCs in carotid artery

**Fig. 3:**
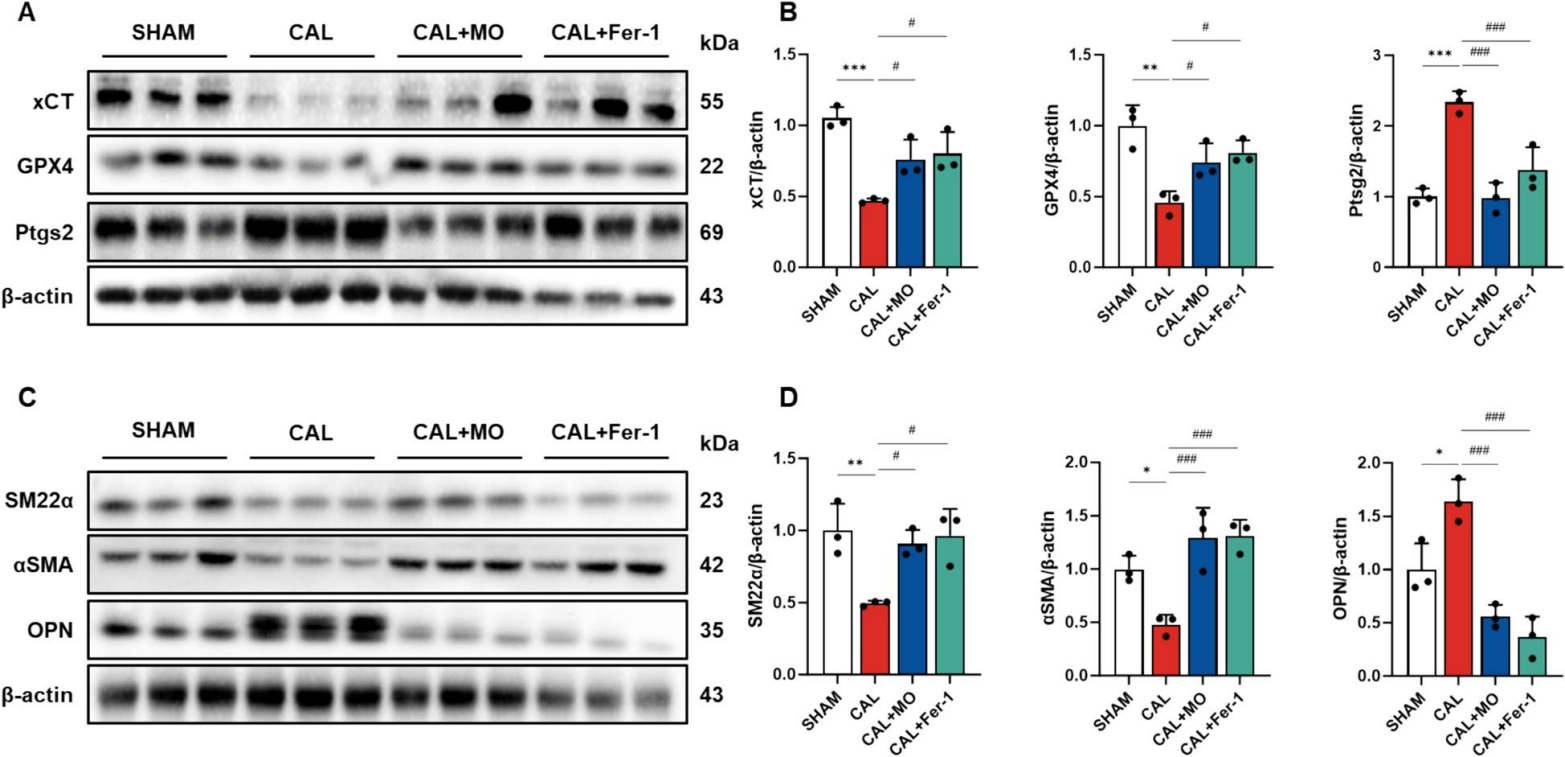
MO suppresses ligation-induced ferroptosis and smooth muscle protein activation in mouse carotid arteries. **A** Western blot images related to ferroptosis in carotid artery. **B** Protein expression fold changes of β-actin. **C** Western blot images related to smooth muscle proteins in carotid artery. **D** Protein expression fold changes of β-actin. Results are represented as mean ± SEM. * *P* < 0.05, ** *P* < 0.01, *** *P* < 0.001 compared to the SHAM group. ^#^ *P* < 0.05, ^###^ *P* < 0.001 compared to the CAL group

Ferroptosis induced by neointimal formation stimulates VSMC proliferation, migration, and phenotypic changes [18]. Era stimulates cellular ferroptosis by inhibiting the expression level of xCT in VSMC [19]. The protective effect of MO against Era-induced ferroptosis and VSMC phenotype switching was evaluated, and the expression of ferroptosis marker protein and contractile proteins related to iron-related ferroptosis and VSMC phenotype are presented in Fig. 3. The ferroptotic protein expression levels of xCT and GPX4 were reduced and expression levels of Ptgs2 were increased in the CAL model (Fig. 3A and B). However, the intake of MO and Fer-1 inhibited ferroptosis. In addition, CAL group reduced the VSMC expression of SM22α, αSMA, and OPN (Fig. 3C and D). However, VSMC protein expression levels of SM22α, αSMA, and OPN were significantly ameliorated by the consumption of MO and Fer-1 compared to the CAL group.

### MO recovers antioxidant system damage in carotid artery

**Fig. 4:**
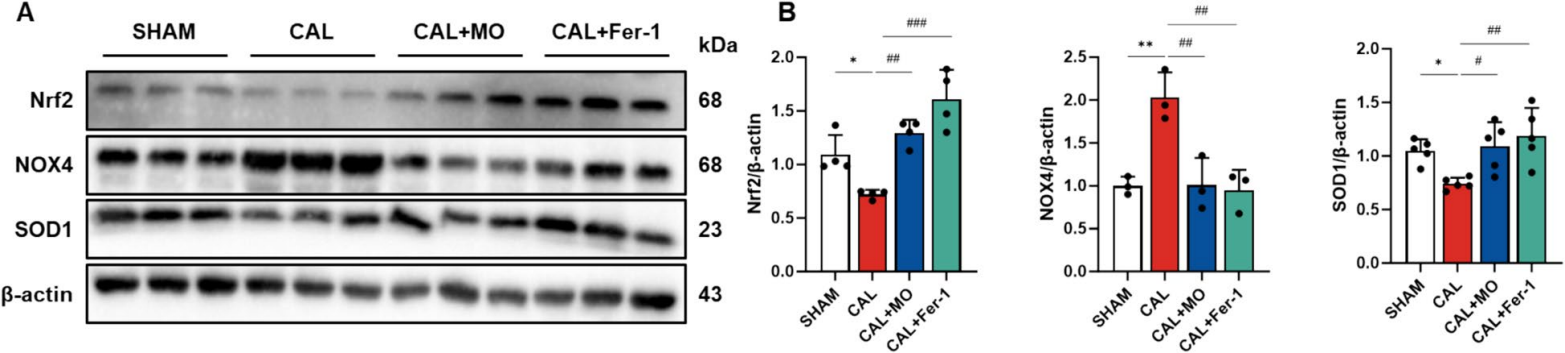
MO suppresses ligation-induced antioxidant system damage in mouse carotid arteries. **A** Western blot images related to antioxidant system in carotid artery. **B** Protein expression fold changes of β-actin. Results are represented as mean ± SEM. * *P* < 0.05, ** *P* < 0.01 compared to the non-Era treatment group. ^#^*P* < 0.05, ^##^*P* < 0.01, ^###^*P* < 0.001 compared to the CAL group

Antioxidant defense systems are important for protecting the cellular walls of vessels and regulating vascular elasticity [20]. To measure the protective effect of MO against ferroptosis, the expression levels of proteins related to the antioxidant system were evaluated (Fig. 4). The protein expression levels of Nrf2 and SOD1 decreased in CAL group, while the protein expression level of NOX4 increased. Therefore, the administration of MO suppressed neointimal hyperplasia by regulating VSMC phenotypic switching, thereby mitigating ferroptosis (Figs. 2, 3, 4).

### MO has inhibitory effect against Era-induced oxidative stress in A7r5 cells

**Fig. 5:**
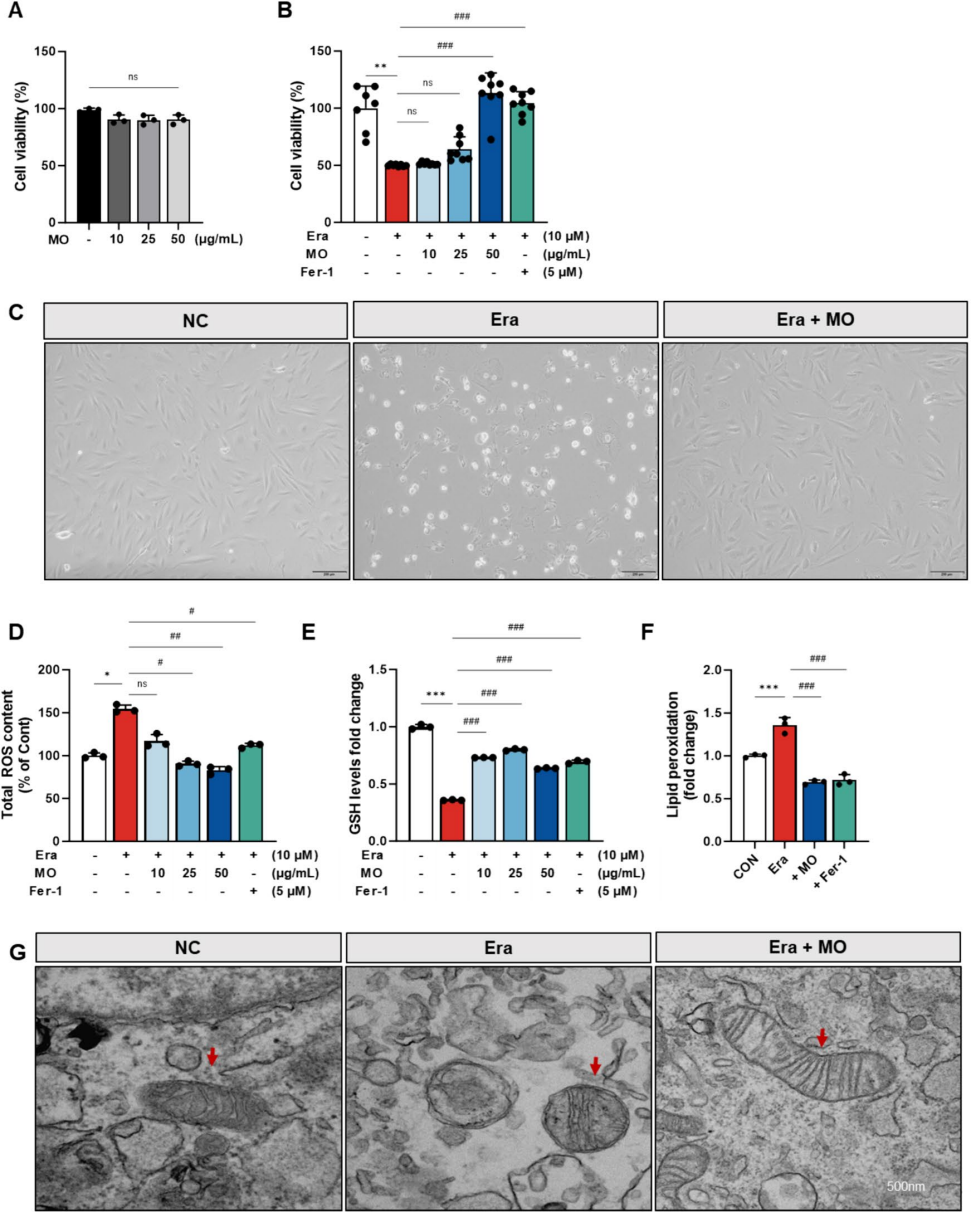
MO suppresses ROS stress in Era-induced A7r5 cells. **A** Cell viability of MO without Era treatment. **B** Cell viability of MO with Era treatment. **C** Microscope images of Era-induced A7r5 cells. **D** Total ROS content (% of control). **E** GSH levels (fold change). **F** Lipid peroxidation (fold change). **G** TEM images of Era-induced A7r5 cells. The red arrows indicate cellular mitochondria. Results are represented as mean ± SEM. *P < 0.05, **P < 0.01, ***P < 0.001 compared to the non-Era treatment group. ^#^*P* < 0.05, ^##^*P* < 0.01, ^###^*P* < 0.001 compared to the Era treatment group. ns indicates no significant difference between groups

Ferroptosis is a specific pathway associated with various diseases of VSMCs [3]. To establish an in vitro ferroptosis model, Era, a ferroptosis activator, was used to induce ferroptosis in A7r5 cells. To investigate the cellular protective effect of MO, MO and Fer-1 were treated in conjunction with Era in A7r5 cells (Fig. 5). Under basal conditions, MO treatment showed no cytotoxic effects up to 100 μg/mL. However, Treatment with MO and Fer-1 significantly recovered cell viability against Era-induced cell death (Fig. 5A and B). Microscopic morphological changes, including cell shrinkage and cell membrane irregularities, were observed in the Era-induced group (Fig. 5C). In contrast, these morphological abnormalities were improved by MO treatment. In addition, treatment with MO and Fer-1 significantly recovered ROS levels, GSH levels, and lipid peroxidation against Era-induced cellular ferroptosis (Fig. 5D–F). TEM analysis presented mitochondrial damage characterized by swelling, cristae loss, and outer membrane deficit in the Era group. However, MO treatment inhibited ferroptosis-induced mitochondrial dysfunction (Fig. 5G).

### MO suppresses Era-induced VSMC proliferation and migration in A7r5 cells

**Fig. 6:**
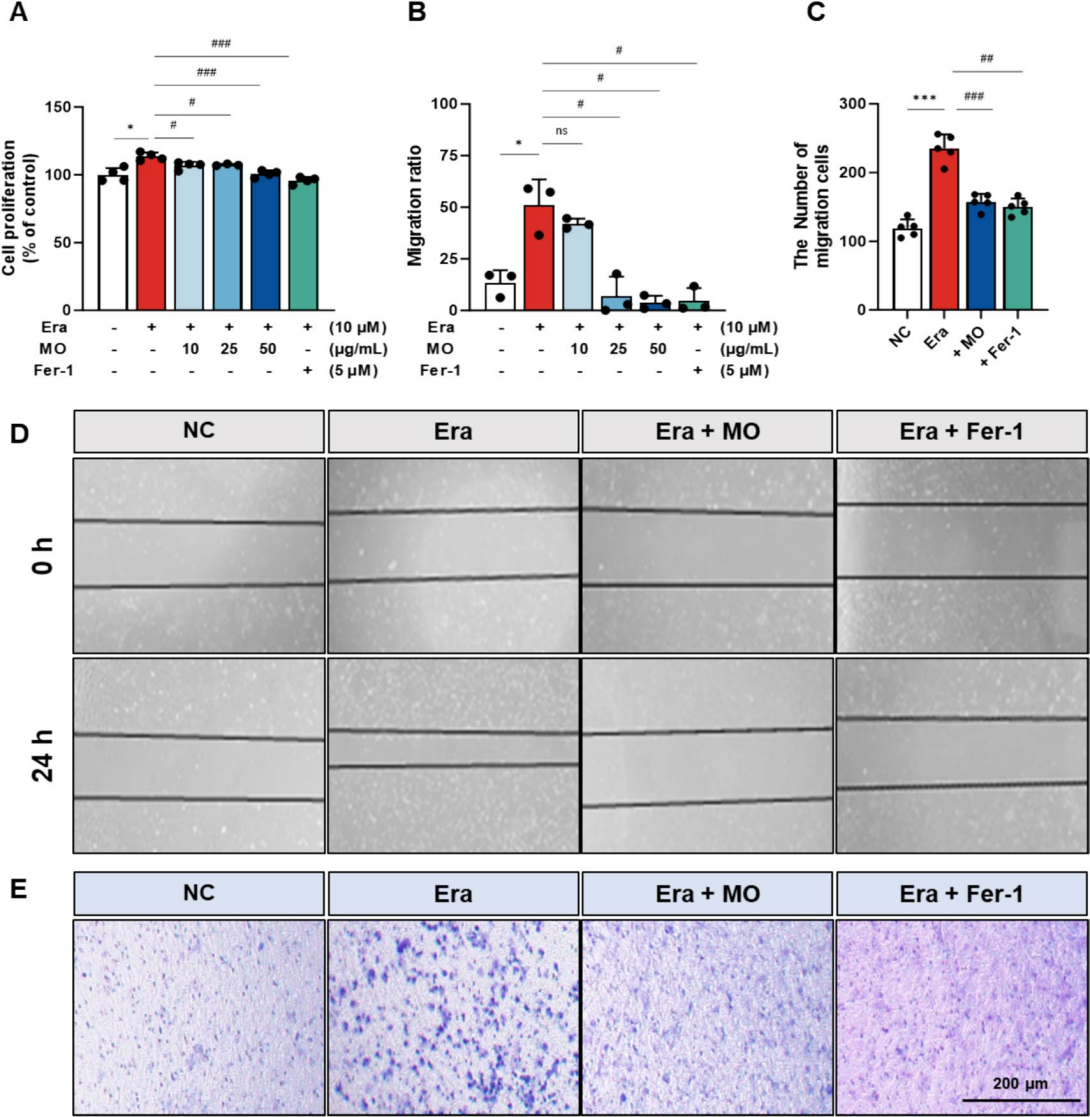
MO suppresses proliferation and migration in Era-induced A7r5 cells. **A** Proliferation evaluation using the BrdU assay. **B** Quantification of migration distance. **C** Quantitative analysis of migrating cells. (**D**) Wound-healing assay at 0 and 24 h. **E** Transwell assay of cell migration capacity. Results are represented as mean ± SEM. *P < 0.05, ***P < 0.001 compared to the non-Era treatment group. ^#^*P* < 0.05, ^##^*P* < 0.01, ^###^*P* < 0.001 compared to the Era treatment group. ns indicates no significant difference between groups

To evaluate the inhibitory effect of MO on Era-induced VSMC proliferation, cell proliferation and migration were measured in A7r5 cells (Fig. 6). MO treatment significantly decreased the number of migrated cells compared to the Era group. Cell proliferation was significantly increased in the Era-treated group compared to the NC group (Fig. 6A). Treatment with MO and Fer-1 significantly reduced cell proliferation compared to the Era group. In the wound healing assay, the migration area in the Era-treated group was significantly increased compared to the NC group (Fig. 6B and D). However, MO and Fer-1 treatments significantly suppressed the migration area compared to the Era group. Similarly, in the transwell migration assay, the number of migrated cells in the Era group increased compared to the NC group (Fig. 6C and E). However, MO and Fer-1 treatment significantly decreased the number of migrated cells compared to the Era group.

### MO regulates Era-induced ferroptosis in A7r5 cells

**Fig. 7:**
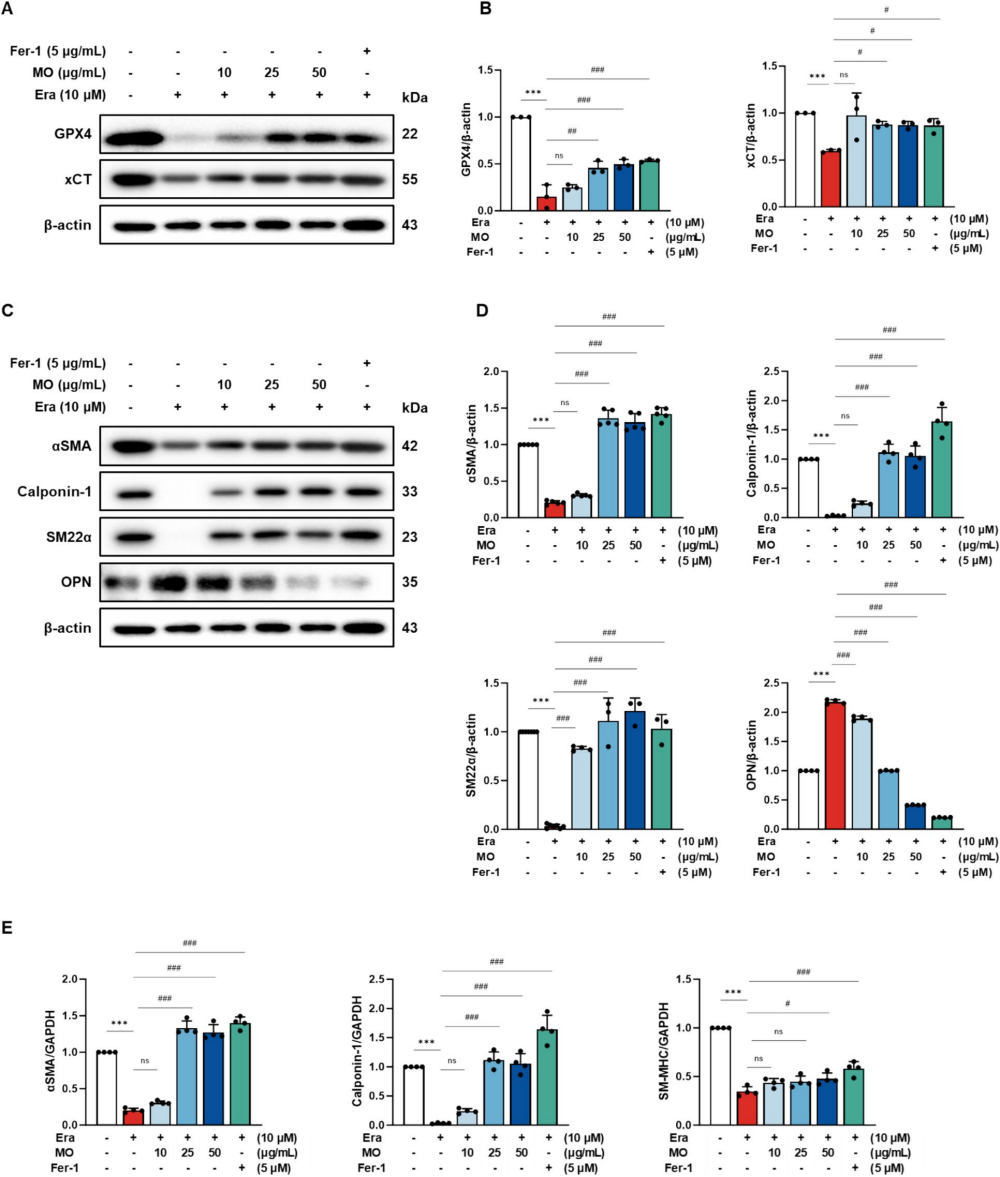
MO suppresses ferroptosis and smooth muscle proteins in Era-induced A7r5 cells. **A** Western blot images related to ferroptosis in Era-induced A7r5 cells. **B** Protein expression fold changes of β-actin. **C** Western blot images related to smooth muscle proteins in Era-induced A7r5 cells. **D** Protein expression fold changes of β-actin. **E** mRNA expression related to smooth muscle proteins fold changes of GAPDH. Results are represented as mean ± SEM. ***p < 0.001 compared to the non-Era treatment group. ^#^*P* < 0.05, ^##^*P* < 0.01, ^###^*P* < 0.001 compared to the Era treatment group. ns indicates no significant difference between groups

To estimate the regulatory effect of MO against Era-induced ferroptosis, the protein expression levels of the ferroptosis markers GPX4 and xCT were measured in A7r5 cells (Fig. 7A and B). The protein expression levels of GPX4 and xCT were lower in the Era-treated group compared to those in the normal control (NC) group. However, the ferroptotic protein expression levels were significantly increased by treatment with MO and Fer-1.

### MO alleviates Era-induced phenotype change of VSMCs in A7r5 cells

To assess the ameliorating effect of MO against Era-induced VSMC dysfunction, the protein expression of αSMA, calponin-1, SM22α, and OPN, as well as the mRNA expression of smooth muscle myosin heavy chain (SM-MHC), αSMA, and calponin-1 were estimated in Era-induced A7r5 cells (Fig. 7C–E). The protein expression levels of αSMA, calponin-1, and SM22α, as well as the mRNA expression levels of SM-MHC, αSMA, and calponin-1, in the Era-induced group were downregulated compared to the NC group. However, MO and Fer-1 significantly suppressed VSMC damage during Era-induced ferroptosis.

### MO decreases Era-induced antioxidant system damage in A7r5 cells

**Fig. 8:**
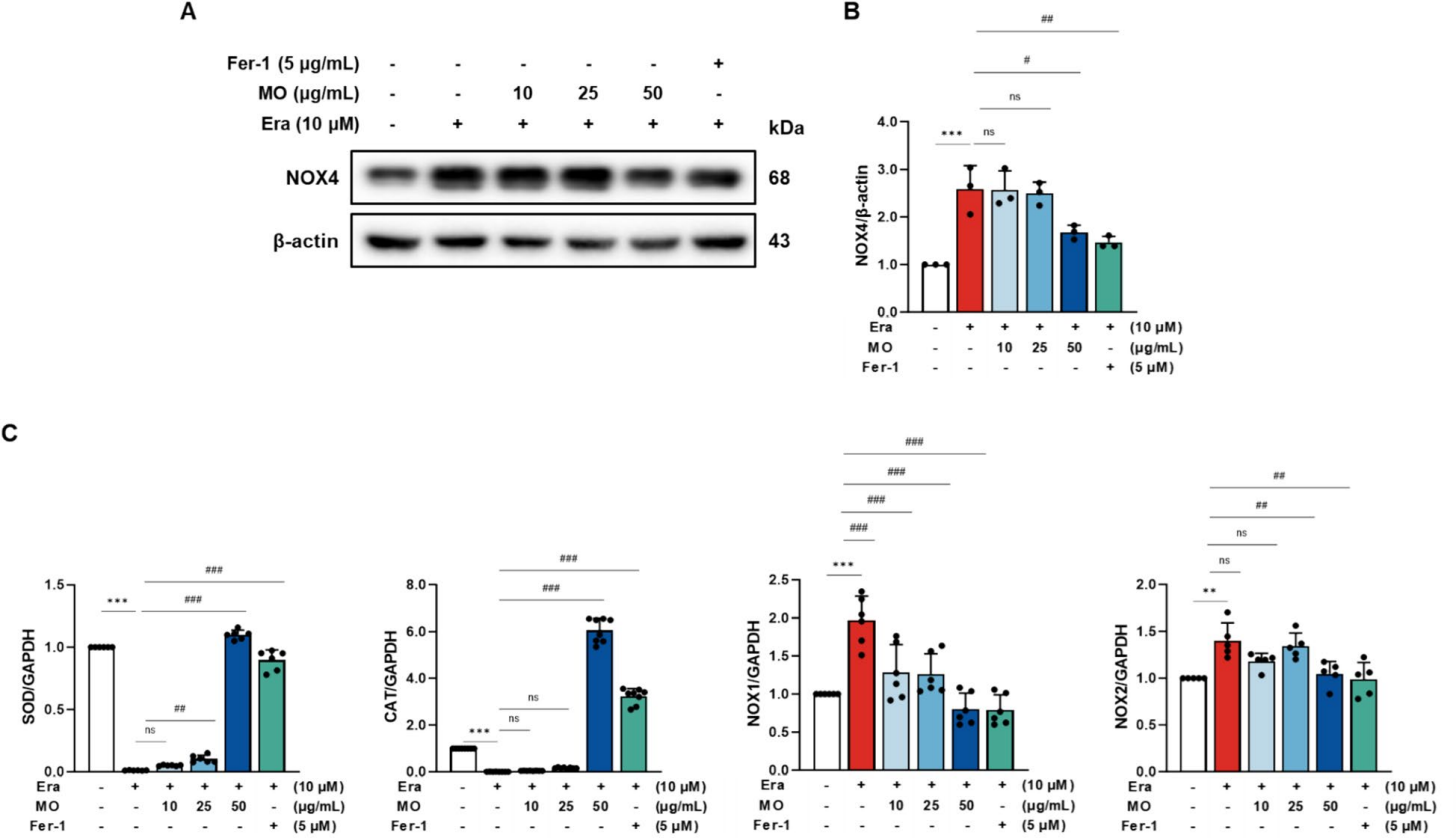
MO reverses antioxidant system damage in Era-induced A7r5 cells. **A** Western blot images related to antioxidant system in Era-induced A7r5 cells. **B** Protein expression fold changes of β-actin. **C** mRNA expression related to antioxidant system fold changes of GAPDH. **P < 0.01, ***P < 0.001 compared to the non-Era treatment group. ^#^*P* < 0.05, ^##^*P* < 0.01, ^###^*P* < 0.001 compared to the Era treatment group. ns indicates no significant difference between groups

To evaluate the protective effect of MO against Era-induced antioxidant system damage, the protein expression levels of NOX4 and the mRNA expression levels of SOD, catalase (CAT), NOX1, and NOX2 were evaluated in Era-induced A7r5 cells (Fig. 8). The protein expression levels of NOX4 and mRNA expression levels of NOX1 and NOX2 in the Era-induced group were significantly upregulated compared to those in the NC group. In addition, the mRNA expression levels of SOD and CAT were considerably downregulated compared to those in the NC group. However, treatment with MO and Fer-1 significantly ameliorated the antioxidant deficiency in Era-induced A7r5 cells.

### MO has significant anti-ferroptosis effect compared to N-acetylcysteine (NAC)

**Fig. 9:**
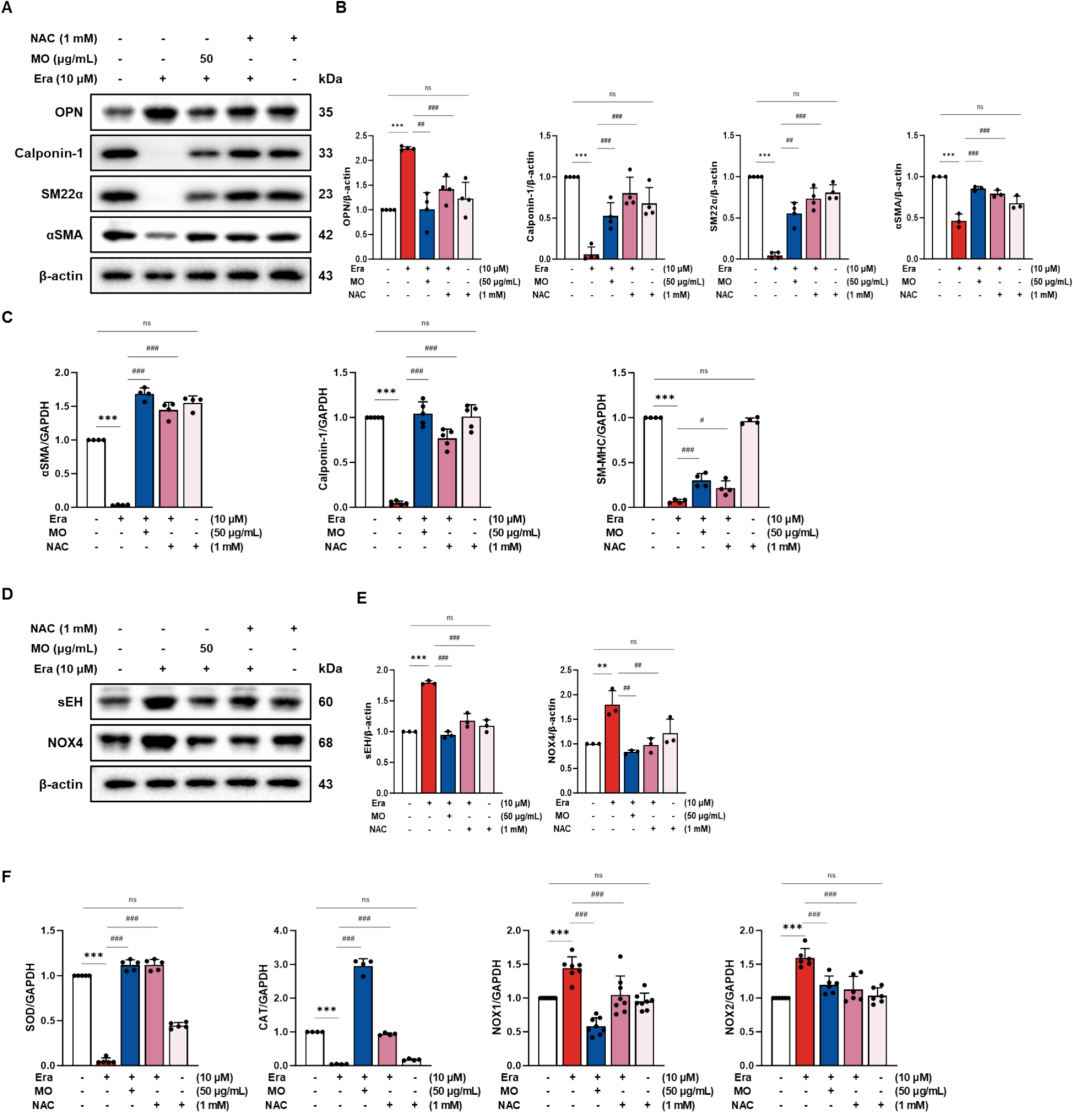
MO suppresses smooth muscle proteins and antioxidant system damage in Era-induced A7r5 cells (comparison with NAC as ROS scavenger). **A** Western blot images related to smooth muscle proteins in Era-induced A7r5 cells. **B** Protein expression fold changes of β-actin. **C** mRNA expression related to smooth muscle protein fold changes in GAPDH. **D** Western blot images related to antioxidant system damage in Era-induced A7r5 cells. **E** Protein expression fold changes of β-actin. **F** mRNA expression related to antioxidant system fold changes in GAPDH. *P < 0.05, ***P < 0.001 compared to the non-Era treatment group. ^#^*P* < 0.05, ^##^*P* < 0.01, ^###^*P* < 0.001 compared to the Era treatment group. ns indicates no significant difference between group

To assess the regulatory effect of MO on VSMCs and the ROS scavenging system, additional studies were conducted by comparing MO with NAC, a ROS scavenger that contributes to the donation of GSH (Fig. 9). The protein expression levels of OPN, sEH, and NOX4, as well as the mRNA expression levels of NOX1 and NOX2, in the Era-induced group were significantly upregulated compared to those in the NC group. The protein expression levels of calponin-1, SM22α, and αSMA, and the mRNA expression levels of calponin-1, αSMA, SM-MHC, SOD, and CAT in the Era-induced group were significantly downregulated compared to those in the NC group. In contrast, MO treatment significantly recovered ferroptotic antioxidant system damage, similar to NAC.

## Discussion

Neointimal formation is the development of new tissue within the inner vascular wall and is closely associated with vascular diseases [21]. Although various causes of intravascular damage have been reported, only a few studies have investigated the role of ferroptosis. Ferroptosis causes cell damage and cell death and affects VSMC signaling. During ferroptosis, VSMCs are influenced by the excessive accumulation of iron ions and lipid peroxidation. This leads to changes in the state and function of VSMCs, contributing to neointima formation [2]. Ferroptosis reduces antioxidant substances, such as GSH and cysteine, and increases cellular oxidative stress [22]. This may elevate oxidative stress within the vascular wall in VSMCs, thus promoting neointimal formation and reducing vascular wall stability [23]. Ferroptosis induced by neointimal formation can trigger cell proliferation and migration, playing a crucial role in the early stages of neointimal formation and resulting in abnormal changes in the vascular structure [24]. The precise sequence is complex, however, microdamage to the vascular wall typically occurs first, followed by inflammation, tissue regeneration processes, and the progression of neointimal hyperplasia in endothelial cells and VSMCs within the vascular wall [25]. Therefore, this study was conducted to evaluate the protective effects of MO using a model that promotes neointimal formation through artificial carotid artery damage-induced ferroptosis.

In this study, neointimal formation was confirmed in the CAL mice model, and the thickness of the intima and the PCNA expression levels observed in H&E staining were significantly increased (Fig. 1). In contrast, MO supplement effectively inhibited neointimal hyperplasia of the carotid artery. These results suggest that MO can be used as a potential material to inhibit neointimal formation and the initiation of proliferation. Neointima formation occurs for various reasons. It has been reported that the induction of ferroptosis, VSMC phenotypic changes, and disruption of the antioxidant system continuously cause vascular diseases derived from damage to vessels and neointimal hyperplasia [22]. These factors are closely related to each other. Because the results of this study were topical results obtained from a surgical animal model, there are limitations to evaluating the effects on the whole body. However, if this phenomenon occurs throughout the body, it can cause various diseases, including cardiac disease, atherosclerosis, aneurysms, and dementia [26], thus making it important to address this phenomenon in advance through the intake of functional materials [27]. In the present study, MO containing various bioactive compounds (Fig. 1) showed significant activity. In particular, caffeoyl quinic acid (CQA) derivatives in MO have neuroprotective effects, and their antihypertensive activity is derived from their considerable antioxidant activity [28–30]. Di-CQA isomers and chlorogenic acid exert vasoprotective effects against high-fat diets and angiotensin-induced cytotoxicity [31, 32]. In addition, the protective effects of MO may be similar to those of Fer-1, an inhibitor of ferroptosis. Therefore, MO can be effectively used to prevent neointimal hyperplasia.

GPX4 plays an important role in vascular cells [33], including GSH production through the transport of glutamine and cysteine into cells via xCT [34]. The abnormal function of this system might induce lipid peroxidation and oxidative stress, which can damage lipids within the blood vessel walls and cause inflammation [35]. This dysfunction causes blood vessel walls to thicken, making it difficult for blood to flow within them, which can be associated with atherosclerosis, high blood pressure, and blood clot formation [36]. Therefore, the abnormal expression of these proteins ultimately damages vascular function through lipid peroxidation and excessive iron accumulation. This pathway is important for maintaining cellular redox balance, and oxidative stress resulting from altered expression of GPX4 may cause severe vascular dysfunction in VSMC [33]. Through the CAL model, we confirmed that vascular dysfunction induces abnormalities in the GPX4/xCT pathway. Abnormal changes in these pathways impair the GSH metabolic balance in VSMC, ultimately leading to ferroptosis, impaired antioxidant systems, and altered changes in VSMC phenotype [25]. On the other hands, MO containing various compounds recovered the GPX/xCT pathway in neointimal formation-induced mice (Fig. 3). Additionally, to evaluate the protective effect of MO, the inhibitory effect against Era-induced ferroptosis was measured in A7r5 cells (Fig. 5). Era inhibits the expression of xCT by blocking the cystine into cells and induces cellular ferroptosis [19]. This reduces GSH levels, leading to the ROS production, mitochondrial dysfunction, and cell death. However, treatment of MO significantly inhibited Era-induced ferroptosis in A7r5 cells. In one study, 3,5-di-CQA was shown to suppress mitochondrial dysfunction and ferroptosis by regulating the expression of GPX4, ACSL4, and SLC7A11 in colorectal cancer cells [37]. In addition, according to Jung et al. [38], MO exerts a neuroprotective effect against glutamate-induced oxidative stress via the regulation of the GSH system, including the modulation of GSH levels, GSH reductase and GSH peroxide activity, as well as intracellular ROS and Ca^2+^ levels in hippocampal cells. Similar to these previous studies, bioactive compounds containing MO, such as CQA derivatives, may exert a protective effect against various ferroptotic cytotoxic actions through the GPX4/xCT-related signaling pathway.

VSMC plays an important role in maintaining vascular integrity and function [2]. Phenotypic regulation of VSMCs, switching from a contractile to a synthetic phenotype, is important in the pathogenesis of various vascular diseases, including atherosclerosis and neointimal hyperplasia [21]. This transition is often accompanied by changes in cell proliferation, migration, and extracellular matrix production, which are key contributors to vascular remodeling. In this study, the expression of SM22α and αSMA, markers of the contractile phenotype, was significantly down-regulated under ferroptotic conditions (Fig. 3B). Conversely, the expression of OPN, a marker of the synthetic phenotype, was upregulated. These findings suggest that ferroptosis not only induces cell death but also drives the phenotypic switch in VSMC, which is associated with increased proliferative and migratory capabilities. In contrast, MO intake significantly ameliorated their expression. Additionally, in the results of cellular studies, Era treatment under ferroptosis conditions increased vascular migration. Increased migration activity of VSMCs leads to thickening of the vascular wall and neointimal hyperplasia [19]. However, treatment with MO significantly reduced this migration, suggesting that MO can effectively inhibit pathological migration of VSMC and prevent excessive vascular remodeling. VSMC phenotype changes induced by ferroptosis were also effectively improved by MO treatment (Fig. 7C–E). In summary, this study demonstrates that MO presented a protective effect on VSMC by both preserving their contractile phenotype and inhibiting their migratory activity. These findings suggest that MO has the potential to prevent various vascular diseases induced by ferroptosis.

Regulation of oxidative stress in VSMCs is important for cellular survival and function, particularly in ferroptotic conditions [22]. Ferroptosis resulting from iron accumulation and lipid peroxidation seriously impairs the antioxidant defense system in these cells, causing oxidative damage and influencing the progression of various vascular diseases [11]. In this study, the expression of antioxidant enzymes and oxidative stress markers was significantly altered under ferroptotic conditions (Fig. 4). In the CAL model, the expression of Nrf2, a key regulator of antioxidant response, was down-regulated, whereas the expression of NOX4, a pro-oxidant enzyme, was up-regulated. The reduction in Nrf2 levels is related to decreased transcription of SOD1, neutralizing superoxide radicals, further increasing oxidative stress within the cells [24]. This imbalance between oxidative stress and antioxidant defenses exacerbates cellular damage in VSMCs, promoting conditions like atherosclerosis and neointimal hyperplasia [33]. However, treatment with MO significantly recovered the expression of these markers. These results are further supported by the increased expression levels of oxidative stress markers such as NOXs under ferroptosis conditions, indicating increased ROS production (Fig. 8). However, MO treatment significantly decreased the expression of NOX1 and NOX4, and upregulated antioxidant enzymes such as SOD and CAT. These changes suggest that MO not only reduces ROS production but also enhances the antioxidant capacity of VSMCs, thereby protecting them from oxidative damage induced by ferroptosis.

Additionally, to evaluate whether ferroptosis-related vascular damage could be prevented through the protection of the antioxidant system, MO was compared with NAC, a ROS scavenger [39]. NAC treatment significantly reduced ROS damage in VSMCs. Similarly, treatment with MO exhibited regulatory effects on antioxidant-related mechanisms against Era-induced oxidative stress and a protective effect on the activity of the antioxidant system by regulating ferroptotic marker expression, VSMC phenotypic changes, and ROS scavenging markers (Fig. 9). MO treatment showed relatively superior protection of contractile phenotype markers such as SM22α and αSMA compared to NAC. This suggests that MO is more effective in preventing the phenotypic switch from a contractile to a synthetic state, which is an important factor in vascular remodeling and disease progression. The increased expression of OPN, a marker of the synthetic phenotype, was significantly reduced with MO treatment, indicating its potential to maintain VSMCs in their functional, contractile state. Moreover, MO demonstrated superior efficacy in modulating the expression of antioxidant enzymes. The expression levels of SOD and CAT were significantly higher in MO-treated cells than in those treated with NAC. This suggests that MO not only scavenges ROS but also enhances the cellular antioxidant capacity more effectively than NAC. Additionally, MO was more effective in downregulating the expression of NOX enzymes, which are major sources of ROS production in VSMC. The inhibition of NOX enzymes by MO further supports its role in reducing oxidative stress and protecting VSMCs from ferroptosis-induced damage. Therefore, these results suggest that supplementation with MOs with excellent antioxidant activity may help regulate vascular abnormalities by improving the antioxidant system.

## Conclusions

In conclusion, this study suggests that MO, containing various bioactive compounds, protects against neointima formation by regulating ferroptotic progression and VSMC phenotypic changes in CAL mice. MO administration significantly suppressed neointima formation in a mouse ligation model. It downregulated ferroptosis induction, mitigated abnormal VSMC changes, and protected against antioxidant system damage in the CAL mouse model and in A7r5 cells. In addition, MO significantly suppressed ferroptosis-induced abnormal cell status by regulating cell viability, ROS scavenging activity, GSH levels, lipid peroxidation, proliferation, and cell migration in Era-induced A7r5 cells. In conclusion, these findings highlight the mutual influence between ferroptosis and vascular dysfunction, emphasizing the vasoprotective effects of MO against CAL-induced neointimal status and Era-induced abnormalities in VSMCs. However, few studies have revealed the individual vascular protective effects of bioactive materials contained in MO. Therefore, additional studies should be conducted on the physiological activity of each compound against ferroptosis-induced vascular dysfunction. In addition, it is necessary to identify various unidentified compounds further and detect various bioactive substances.

## Data Availability

The data sets used and/or analyzed during the current study are available from the corresponding author on reasonable request.
