# Correction: *Magnolia kobus* DC. suppresses neointimal hyperplasia by regulating ferroptosis and VSMC phenotypic switching in a carotid artery ligation mouse model

**DOI:** 10.1186/s13020-025-01066-5

**Published:** 2025-02-18

**Authors:** Jong Min Kim, Yiseul Kim, Hyun‑Jin Na, Haeng Jeon Hur, Sang Hee Lee, Mi Jeong Sung

**Affiliations:** Aging and Metabolism Research Group, Korea Food Research Institute, Wanju‑gun, 55365 Republic of Korea

**Correction: Chinese Medicine (2025) 20:3** 10.1186/s13020-024-01051-4

Following publication of the original article [1], it is reported that the corresponding author’s name was incorrect. The corresponding author’s name is corrected from Sung to Mi Jeong Sung.

The original article [1] has been updated.
